# Anterior Tooth Replacement of Avulsed Deciduous Tooth: Resin-Reinforced Fiber With Natural Tooth Pontic

**DOI:** 10.7759/cureus.19605

**Published:** 2021-11-15

**Authors:** Mansi Gandhy, Ajit Baviskar

**Affiliations:** 1 Pediatric Dentistry, DY Patil School of Dentistry, Navi Mumbai, IND; 2 Emergency Medicine, DY Patil School of Medicine, Navi Mumbai, IND

**Keywords:** pediatric dental trauma, young child, resin reinforced fibre, natural pontic, avulsed deciduous anterior tooth

## Abstract

Sudden tooth loss can be a traumatic experience that affects a child psychologically and hampers social skills in today’s world, in which appearances seem to matter from a young age. The child becomes afraid to smile freely. Besides appearances, missing anterior teeth can hamper clarity in speech. Many approaches have been described in the past, such as the use of a removable partial denture, functional Nance appliance, Groper’s appliance, and resin-reinforced fiber composite/acrylic pontic. We report the case of a child who lost a deciduous tooth as a result of trauma. The restoration was performed using fiber-reinforced resin and a natural avulsed tooth as pontic, which restored esthetics and function.

## Introduction

The early loss of anterior teeth can have a debilitating effect on a child’s psyche. In today’s world, where looks play a major role in being accepted by peers, a sudden loss of a tooth due to trauma can result in the loss of confidence in a child. For a three- or four-year-old child, entering preschool is the time to make friends and be accepted by peers. In such a situation, the loss of anterior teeth can lead to being teased or made fun of, which results in the child being withdrawn and refusing to smile [1].

Replacing the anterior tooth becomes mandatory for the psychological wellbeing of the child. Many appliances such as the removable partial denture, functional Nance appliance, and fiber-reinforced composite pontic have been described [2-4], with the former being a removable appliance and the latter being fixed appliances. The use of the fiber-reinforced pontic is a relatively recent addition to achieve space maintenance, strength of functionality, and uncompromised esthetics. A step ahead would be to use the avulsed tooth as a pontic [5,6]. This would undoubtedly have enhanced esthetics and strength.

The reinforcement of a composite with fiber improves its fracture toughness and resistance [7]. The glass-fiber-reinforced composite material offers a minimally invasive, metal-free, cost-effective, and esthetic option for tooth replacement. Besides the ease of procedure with no laboratory time, non-bulky prosthesis, and less chair time, it is also convenient for a little child with little additional maintenance and the added advantage of impeccable esthetics.

This case report describes the use of an avulsed natural deciduous anterior tooth as a pontic for a fiber-reinforced composite resin-fixed prosthesis. The authors did not find a similar case report of an avulsed primary natural tooth being used as a pontic to replace a missing tooth with resin-reinforced fiber in the literature.

## Case presentation

**Figure 1:**
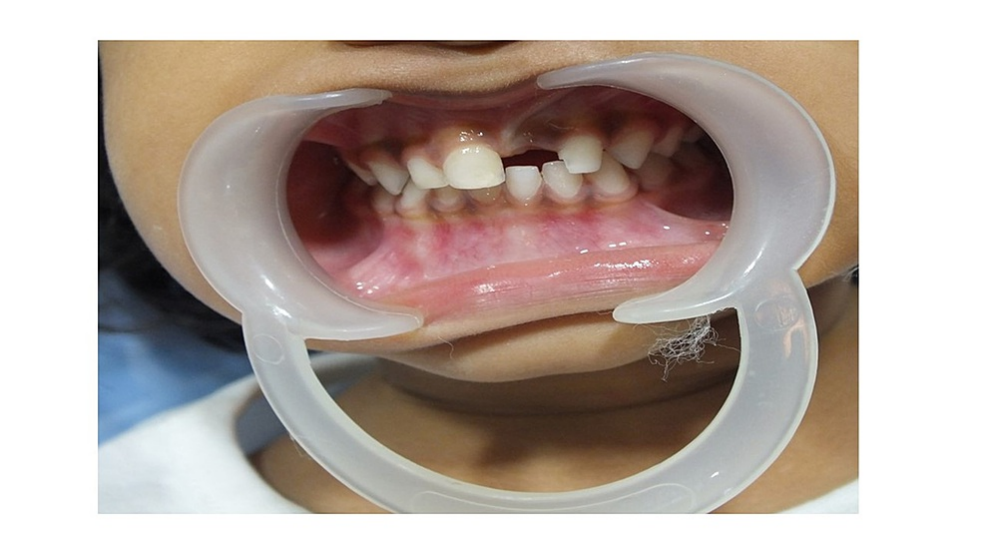
Three-and-a-half-year-old child with avulsed 61

A mother brought in her three-and-a-half-year-old daughter with a month-long history of avulsion. On examination, the anterior upper left central incisor was missing (Figure 1).

The region of 61 showed healing. No other oral findings or cavities were seen. Oral hygiene was good. An intraoral periapical radiograph was taken to rule out a broken tooth or intrusion. Coincidentally, the mother had kept the avulsed tooth as a souvenir. The mother was concerned about the child's looks as she was about to start preschool and was keen on replacement. The child, too, was excited about the new tooth and was cooperative.

**Figure 2:**
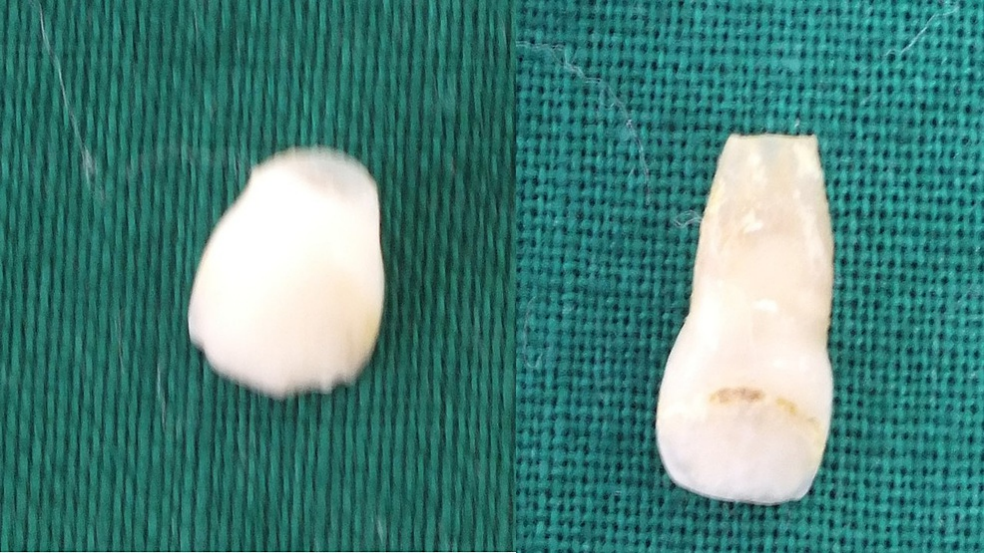
Natural avulsed tooth (right) and the tooth after preparation (left)

The avulsed tooth was prepared by removing the root, cleaning the coronal pulp chamber with hypochlorite and saline, and filling it with an opaque A2 (OA2) composite. It was polished using a composite polishing bur. The lingual aspect of the natural avulsed tooth was prepared, and the cingulum was removed to prevent contact with the lower tooth, which reduces the force on the tooth and creates space for the fiber and flowable composite (Figure 2).

**Figure 3:**
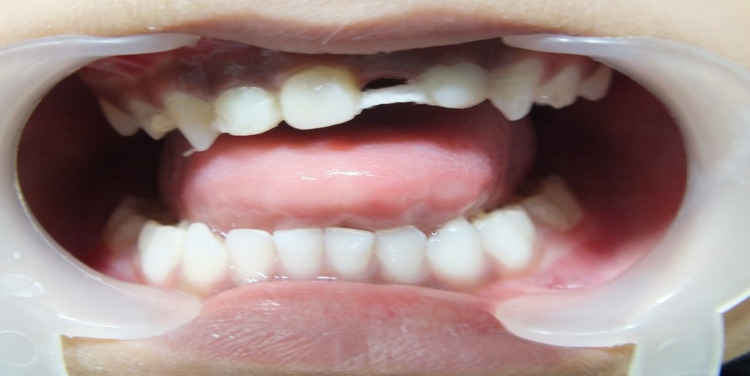
Resin-reinforced fiber material used

The procedure was carried out using cotton rolls and suction for isolation as the child was apprehensive about placing a rubber dam. The neighboring teeth, 51 and 62, were prepared on the palatal surface using an inverted diamond bur (1.5 mm × 1.5 mm × <1 mm) with undercuts for the added surface area and retention of the fiber. The prepared surface was etched with 37% phosphoric acid (Restorite) for 20 seconds, washed, and dried, and it underwent the application of bonding resin (3M single bond universal adhesive). Interlig, a braided glass fiber strip impregnated with light-cure composite resin, was used. The distance between the teeth was measured using dental floss and fiber cut to size. The resin-reinforced fiber was used from 51 to 62, forming a bridge (Figure 3).

**Figure 4:**
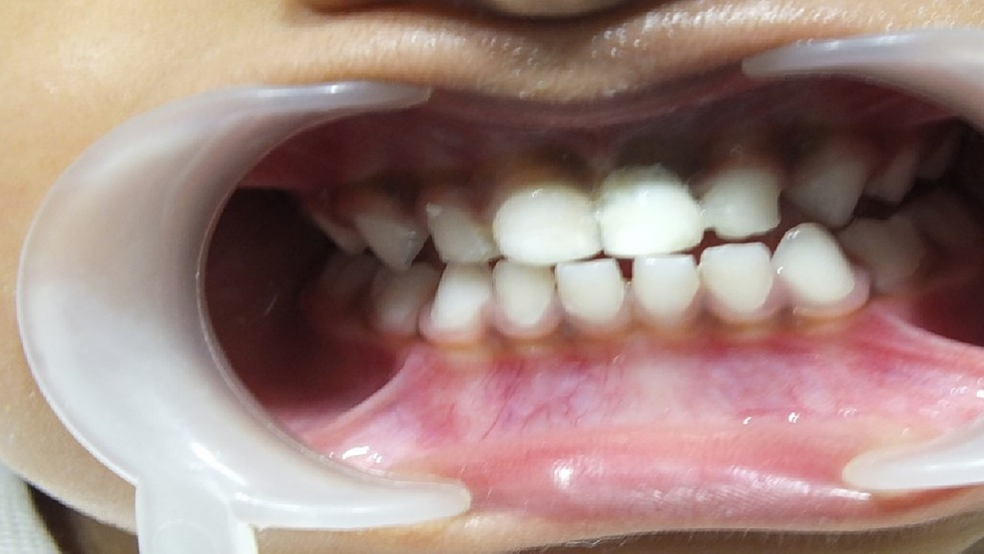
Resin-reinforced fiber cured in position

The lingual aspect of the natural tooth was etched using phosphoric acid for 20 seconds; a bonding agent was applied and cured in position using a flowable composite (A2 G-aenial Universal Flo) (Figure 4).

**Figure 5:**
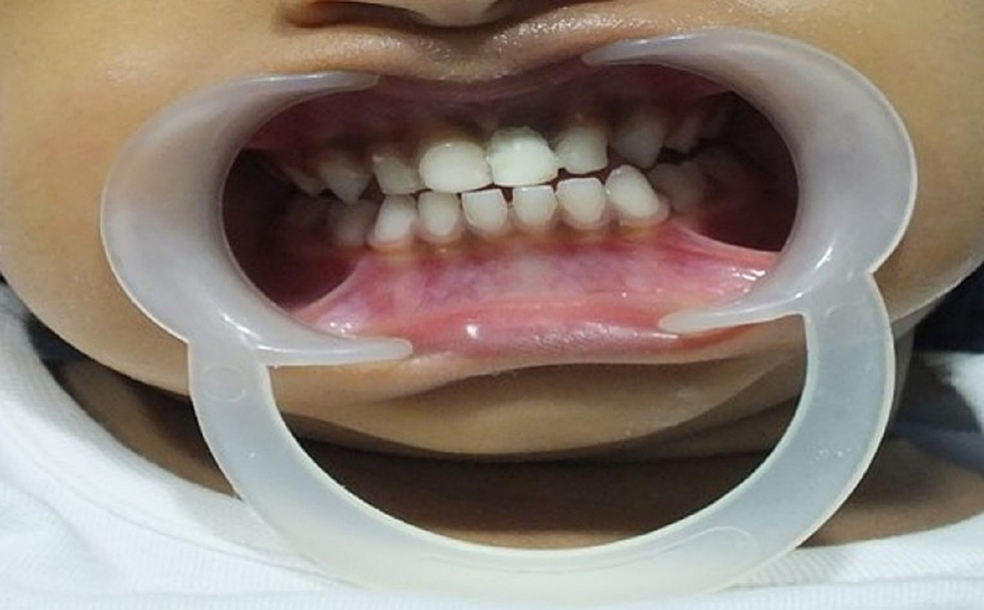
Missing tooth replaced using natural tooth pontic

The entire procedure required approximately 20 minutes of chair time (Figure 5).

The preparation of the avulsed tooth was done prior to the appointment, considering the child's age. The child was asked to avoid using the front teeth to bite and to maintain good oral hygiene.

## Discussion

This clinical case report describes the use of resin-reinforced fiber along with natural avulsed tooth as pontic in patients with deciduous dentition. This was primarily done to restore esthetics and prevent the development of speech defects. The loss of an anterior tooth can have a psychological impact on the child. To prevent this, tooth replacement becomes necessary. Removable functional appliances and Groper’s appliances were not given as they are bulky and require laboratory time and precision [2,3]. The resin-reinforced fiber met the requirement of being non-bulky, conservative, esthetic, and having no metal parts [4]. Since the child had an avulsed tooth, it was decided that the natural tooth be used as a pontic for enhanced esthetics instead of a composite [5,6].

Adjacent teeth were prepared to a depth of <1 mm as deciduous teeth are small, and the thickness of enamel and dentin is less. An inverted bur is used to provide undercuts to increase the surface area for the composite to flow, thereby increasing retention [7]. The composition of the polymer matrix and fiber orientation has a major role in the bonding ability and durability of the prosthesis. It has been concluded that pre-impregnated fibers with light polymerizable dimethacrylate resin systems containing linear polymers form a semi-interpenetrating polymer network after being polymerized, thereby offering more interfacial adhesion of the composite, higher durability, and high strength [8]. Composite reinforced fibers result in higher mechanical strength and toughness and less fatigue [9]. Interlig possesses all the above properties. It is a braided glass fiber strip impregnated with a light-cured composite resin; hence, it was chosen in the case.

The indications for the use of resin-reinforced fiber prosthesis are cooperative children, anterior teeth that do not have to bear heavy masticatory forces, and demand for a less bulky prosthesis that is not made of metal and has a small span. The prosthesis is contraindicated in children with habits like nail-biting and thumbsucking or bruxism, a larger span (three or more teeth), and uncooperative children. An advantage of this prosthesis is the ease of procedure and reparability. It is economical and requires no laboratory time.

## Conclusions

The exfoliation time of the upper anterior deciduous teeth and the subsequent eruption of the permanent anterior teeth occurs approximately at the age of six to seven years. The three-and-a-half-year-old child had a long time before the eruption of permanent anterior teeth. The patient was about to start preschool. Considering these points, replacing the avulsed/missing deciduous tooth with the resin-reinforced fiber-pontic design was a good option. The use of a natural tooth as pontic, if available, can help to enhance the esthetics. This, along with regular follow-up for the prosthesis and oral hygiene maintenance, can help a child gain the required confidence and acceptance during their initial schooling years.
